# Assessment of liver marker enzymes and its association with type 2 diabetes mellitus in Northwest Ethiopia

**DOI:** 10.1186/s13104-019-4742-x

**Published:** 2019-10-29

**Authors:** Tewodros Shibabaw, Gashaw Dessie, Meseret Derbew Molla, Muluken Fekadie Zerihun, Birhanu Ayelign

**Affiliations:** 1 Department of Biochemistry, School of Medicine, College of Medicine and Health Sciences, University of Gondar, Gondar, Ethiopia; 2 Department of Immunology and Molecular Biology, School of Biomedical and Laboratory Science, College of Medicine and Health Sciences, University of Gondar, Gondar, Ethiopia

**Keywords:** Gamma-glutamyltransferase, Aspartate aminotransaminase, Alanine aminotransaminase

## Abstract

**Objective:**

This study aimed to assess the level of aspartate aminotransaminase (AST), alanine aminotransaminase (ALT) and gamma-glutamyltransferase (GGT), and their association with type 2 diabetes mellitus in Northwest Ethiopia.

**Results:**

Using a cross-sectional study, blood samples were collected from 192 Type 2 diabetes mellitus (T2DM) participants and 192 healthy age and sex-matched volunteers. The study was carried out from May to August 2017. The serum concentration of aspartate aminotransaminase, alanine aminotransaminase, and gamma-glutamyltransferase were measured using A25 Bio-system fully automatic chemistry analyzer and using the manufacturer’s kit of the machine. Liver function test results of T2DM participant were significantly higher than those of the control group, serum ALT (46.06 ± 22. 38 IU/L) and serum AST (42.94 ± 19. 08 IU/L), P < 0.001, while the level of GGT in both study groups was not significantly associated (P = 0.065). In conclusion, the evaluation of liver marker enzymes showed a significant association with Type 2 diabetes participants compared with the controls.

## Introduction

Diabetes mellitus is a heterogeneous group of disorders characterized by persistent hyperglycemia with carbohydrate, lipid, and protein metabolism resulting from defects in insulin secretion and/or insulin action [1, 2]. Type 2 diabetes is caused by impaired β-cells function and capacity to secrete sufficient insulin, coupled with a decline in target tissue sensitivity to insulin (insulin resistance) [3, 4]. Globally, type 2 diabetes is one of the most common non-communicable diseases, which is increasing at an alarming rate and affecting a significant number of people. It is rapidly rising as a global health care problem and threatening to reach endemic levels by 2030, especially in low and middle income countries [1, 5, 6].

This metabolic disorder (diabetes) affects many organs, including the liver, which plays a key role in the regulation of carbohydrate, lipid, and protein metabolism [7]. Elevated serum aminotransferases level; Aspartate aminotransferase (AST), alanine aminotransferase (ALT) and γ-glutamyltransferase (GGT) were commonly observed in diabetes [8, 9]. Alanine aminotransferase and aspartate aminotransferase are the most specific marker of hepatic injury, which is located in the hepatocellular cytosol and mitochondria, respectively [10, 11]. A recent report shows a significant association of increased ALT and AST with insulin resistance, T2DM, and metabolic syndrome [9, 12]. Gamma-glutamyltransferase (GGT) is located on the external surface of most cells and mediates the uptake of glutathione, an important component of intracellular antioxidant defenses [13–15]. An increase in GGT concentration has been regarded as a marker of alcohol-associated liver disease than diabetes associated hepatic injury [14]. Even though, diabetes is one of the major public health problems in Ethiopia, there is are highly limited documented and updated articles on the association of diabetes with hepatic injury/dysfunction. Since there is no adequate studies conducted have been to the best of our knowledge, the current study we hope will fill the existing gap in the association of the liver marker enzymes with T2DM. Therefore, we aimed to assess liver enzyme tests, such as ALT, AST and GGT and their association with Type 2 diabetes as compared with non-diabetes control groups in north west Ethiopia.

## Main text

### Materials and methods

#### Study design, sampling technique, and sample size determination

A cross-sectional study was conducted at north west Ethiopia from May to August 2017. A single population proportion formula was used to calculate the sample size, n = Z^2^ p (1 − p)/d^2^, where: Z = Z score at 95% confidence interval = 1.96 with power = 0.80, p = prevalence = 50%, d = mariginal error = 5% (0.05). N = (1.96)^2^ 0.5 (1 – 0.5)/(0.05)^2^ = 384, Finally, a total of 384 study participants were selected by using the simple random sampling technique. We nominated an equal number of cases (diabetes) and control (apparently healthy) study participants, 192 for each group.

#### Inclusion and exclusion criteria

Participants with confirmed diabetes mellitus or newly diagnosed diabetes using WHO guidelines [16], fasting plasma venous glucose of ≥ 7 mmol/L (126 mg/dL) or random plasma venous glucose of ≥ 11.1 mmol/L (200 mg/dL) were included, while exclude the diabetes participants with history of liver disease, alcohol intake, hepatotoxic drugs, clinical evidence for acute hepatitis, participants with hepatitis B and C virus infection, and participants with clinical and subclinical hypothyroidism was excluded.

#### Blood sample collection, analysis and quality assurance

The interviewer was particularly planned to obtain information, which helps to screen participants on the eligibility criteria. Five milliliters (5 mL) of venous blood was drawn from each volunteer participant using disposable plastic syringes. The blood was poured into a plane containers and centrifuged after clotting. Serum was kept at − 20 °C in sterile circumstance at the University of Gondar compressive specialized hospital laboratory until the analysis was done. SGOT, SGPT, and GGT were determined by means of enzymatic tests using the A25 Bio-system human (German). The normal value of each test was based on the reference of enzymatic test of A25 Bio-system human (German) kit. Data quality management/control Training was provided for data collectors and supervisors about the purpose of the study, data collection process, laboratory analysis, and ethical issues. The sample was received, accessioned and processed according to the standard operational procedure. Strict supervision and monitoring were also performed during the data collection period by supervisors and investigators. The standard operational procedure was addressed in the pre-analytic, analytic and post-analytic stages of laboratory services which constituted and impacted the overall quality of the laboratory analysis. Data was also cleaned up and cross checked to control irregularities.

#### Statistical analysis

Data were double entered and cleaned using Epi Data 3.1 (Jens M. Lauritsen & Michael Bruus) and transferred to SPSS version 20 (IBM, New York, and U.S) and presented as mean ± SD values. Statistical analysis was done by using an independent t test to find out the difference between the two unpaired groups. P 0.05 was considered statistically significant.

### Result

A total of 384 participants (192 confirmed T2DM and 192 controls) were enrolled in this study. The mean ages of the T2DM and controls participants were 55.76 ± 10. 11 and 50.93 ± 5.41, respectively. Men constituted 121 (63%) of T2DM participants and 116 (60.4%) of the controls. Sixty-nine (35.9%) of the diabetes case and 54 (28.1%) of controls completed secondary school; 92 (47.9%) of the case and 67 (34.9%) of the controls were merchants and civil servants, respectively (Table 1).

**Table 1:** Socio-demographic characteristics of study participants (N = 384), North West Ethiopia, 2017

Characteristics	Patient (n = 192) N (%)	Control (n = 192) N (%)
Sex		
M	121(63%)	116 (60.4%)
F	71 (37%)	76 (39.6%)
Age (Mean ± SD)	55.76 ± 10.11	50.93 ± 5.41
Religious		
Orthodox	131(68.2)	148 (77.1)
Muslim	35 (18.2)	34(17.7)
Protestant	14 (7.3)	7(3.6)
Catholic	12 (6.3)	3(1.6)
Education		
Illiterate	40 (20.8)	39(20.3)
Primary school	69 (35.9)	77(40.1)
Secondary school and college	69 (35.9)	54(28.1)
Degree and above	14 (7.3)	22(11.5)
Occupation		
Farmer	17 (8.9)	17(8.9)
Merchant	92 (47.9)	79(41.1)
Civil servant	72 (37.5)	64(33.3)
Student	6(3.1)	12(6.3)
Other	5 (2.6)	20(10.4)

*F* Female, *IU/L* International units per liter, *M* male, *mg/dl* milligrams per deciliter, *N* number, *n* number, *SD* standard deviation

**Fig. 1:**
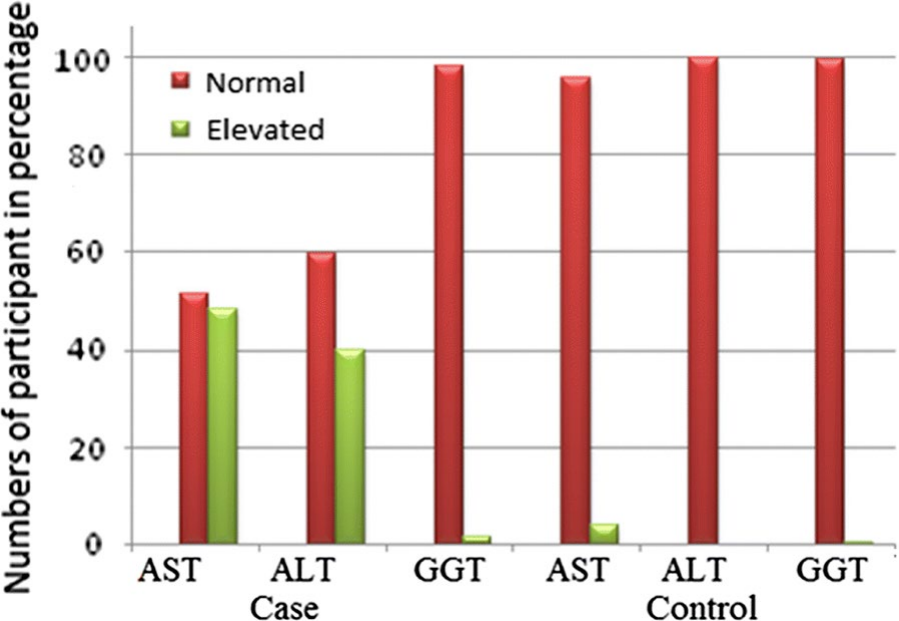
Comparison of liver enzymes among type 2 diabetes mellitus participant and control groups. *AST* Aspartate aminotransaminase, *ALT* alanine aminotransaminase, *GGT* gamma glutamyltransferase

In this study, 93 (48.4%) of diabetes participant and 8 (4.2%) of control had elevated serum AST levels. Of all the diabetes case, 77 (40.1%) had elevated serum ALT, and 192 (100%) of control group had normal values of ALT. In addition, 3 (1.6%) of diabetes case and 1 (0.5%) of the controls had elevated serum GGT levels (Fig. 1).

The mean values of ALT and AST were significantly higher in type 2 diabetes participants than the control group (P < 0.001) as shown in Table 2. The GGT level of the control group and that of the diabetes participants was not associated significantly (P = 0.065). The mean value of fasting blood glucose of in T2DM was significantly higher than that of control group (P ≤ 0.001) as illustrated in Table 2.

**Table 2:** Biochemical parameters among diabetes and control participant (as mean ± SD) (N = 384), Northwest, Ethiopia, 2017

Parameters	DM participants (mean ± SD)	Control group (mean ± SD)	*P* value	Laboratory reference range
FBS(mg/dl)	189.80 ± 64.45	89.52 ± 10.81	< 0.001	60–110 mg/dL
AST(IU/L)	42.94 ± 19.08	20.34 ± 9.90	< 0.001	0–37 U/L
ALT(IU/L)	46.06 ± 22.38	22.66 ± 9.45	< 0.001	0–42 U/L
GGT	21.98 ± 8.36	20.28 ± 9.63	0.065	0–48 U/L

*DM* Diabetes Mellitus, International Units per Liter, *mg/dL* milligrams per deciliter, *SD* standard deviation

### Discussion

In the present study, the mean age of the diabetes and the control group were 56 and 52 years, respectively. Acute and subclinical hepatocellular disturbance might influence plasma glucose homeostasis. Due to the fact that, we can understand that liver enzymes are not the only markers of liver dysfunction, which also have a predictive value to assess the severity of diabetes. Insulin resistance is a typical feature of T2DM, which shows that abnormality in glucose homeostasis by the liver [17]. Glycation is the most common complication of T2DM that results oxidative stress in tissue [18]. This oxidative stress and cytokine production in the liver cause alterations of liver enzymes due to the hepatocellular damage [19, 20]. Thus, further results dysregulation of blood glucose maintenance, since it plays a key role in such maintenance [21]. This condition results in the abnormal introduction of liver enzymes into the circulation and become elevated. In the present study, we have assessed the level of liver enzymes, including ALT, AST, and GGT among age-sex matched T2DM participants and an apparently healthy control groups.

In this study, 93 (48.4%) of the diabetes case and 8 (4.2%) of the control group had elevated serum AST levels. Among the case groups, 77 (40.1%) of them had elevated serum ALT whereas 192 (100%) of the control group had normal values. The result showed that the mean values of AST and ALT significantly increased in T2DM participants compared with the controls group. Our findings were in line with a study conducted in Iraq and India in which the elevation of AST and ALT were statistically associated with T2DM [20, 22]. Likewise, a recent study in Myanmar and Singapore demonstrated that the mean values of ALT and AST were within the reference range among the diabetes participants [23]. In contrast to our finding, the level of GGT was statistically significant with T2DM in the two studys above stated [20]. Elevated ALT and AST value were observed in 18.5% and 14.8 in the T2DM participants, respectively [24]. A similar finding was reported in India, which investigated 90 T2DM for liver enzyme, ALT and 90 healthy subjects as control group [20].

Serum ALT (71.65 ± 23.3) levels were elevated significantly among 36 (40%) of the T2DM participants [10, 24]. Similarly, our result were in agreement with a study conducted on Sudanese diabetes participants and 50 apparently healthy control subjects. The result of this study showed that the mean values of ALT and AST were significantly higher in T2DM than the control group [9, 12]. Even if the mean values were within the normal range, 11 T2DM participants (22%) had at least one or more abnormal liver enzymes [12, 25]. On the other hand, in this study, GGT was not significantly associated with the risk for diabetes. In contrast to this, another study indicated that GGT was significantly associated with the risk among diabetes participants compared to controls [13]. This study suggested that an increase in GGT concentration was a perceptive and an early biomarker of the development of diabetes.

Our study evaluated the presence of elevated levels of liver marker enzymes among T2DM participants. This may be due to increases in glycogen/insulin effect on liver cells. The increase of glycogenolysis (breakdown of stored glycogen) and gluconeogenesis (glucose production from non-carbohydrate precursors) becomes the primary metabolic pathway [26]. Thus, increases in substrate delivery (e.g. alanine) and in the conversion of alanine to glucose might be regulated as a compensatory mechanism for impaired hepatic insulin communication transduction, which allows the enzyme to leak out of hepatocytes, mainly due to the infiltration of fat accumulation as well as hepatic cell injury [23]. An abnormal accumulation of fat and its mobilization in hormone-sensitive tissues (liver) and hepatocytes demonstrate a metabolic switch through insulin resistance identified earlier than the fasting elevation of blood sugar. The overloaded release of free fatty acids due to insulin resistance induces fat mobilization and results in the toxicity of hepatocytes [20]. The elevation of the transaminase enzymes is directly linked to liver cell damages. The rupture of a plasma membrane at high concentrations of the metabolites, loss of mitochondrial activities and the inactivation of the regulatory metabolic enzymes results in hepatic cell injury [27]. In conclusion, the result of this study showed that the elevation of the liver enzyme test (ALT and AST) was significantly associated with type 2 diabetes mellitus compared with the control group. Therefore, liver enzyme tests might have a positive role in the management of diabetes.

## Limitations of the study

The use of limited number of biochemical parameters and the absence of invasive liver biopsy procedures to assess liver histopathological changes is one of the limitation of our work. Further clarification is necessary for large sample studies to exactly illustrate a comprehensive conclusion of liver function tests in a person with T2DM.

## Data Availability

Relevant data used could be made available with in this manuscript.

## Abbreviations

WHO: Health Organization
AST: aspartate aminotransaminase
ALT: alanine aminotransaminase
T2DM: type 2 diabetes mellitus
BMI: body mass index
GGT: gamma glutamyltransferase
